# Our Contemporaries

**Published:** 1915-12

**Authors:** 


### OUR CONTEMPORARIES.

---

Beginning with the January, 1916, number, the Journal of Cutaneous Diseases including Syphilis, will be published for the American Dermatological Association by W. M. Leonard of Boston. Each number will contain 80 pages and as far as possible be of interest and value to the general practitioner as well as to the dermatologist. George M. McKee, M. D., editor.

---

The Medical Summary thinks that propaganda should be issued against THE USELESS DOG, as against flies, mosquitoes and other nuisances. The farm dog easily earns his keep in fetching cows for milking, driving sheep, guarding property and catching woodchucks, not to mention his occasional work on a treadmill for churning, etc. In the city, the case is different, but there is something to be said on the other side. For example, there is a 7-lb. pet, too small to be of any serious use, although he has the fearlessness and spirit of a St. Bernard. Burglars and tramps, as our contemporary states, are not a frequent danger, and such a dog would be powerless to inflict damage on them, but it is worth a few bones and crumbs to feel confident that, day or night, his keener senses will notify of any intruder or of any unusual event of any kind. Probably he does not realize that those bones and crumbs depend pretty directly on attention to the telephone and door bell but, solely of his own mental workings, he has grasped the fact that those signals must be answered, and that persons who enter without ringing must receive attention. By relaying such signals to less acute ears of two-legged persons, he already has a considerable credit balance beyond the modest demands of his stomach. On the basis of unsolicited, bona fide offers, he represents a 400% gain in investment value, though we hasten to add that it would be extremely unwise to take advantage of these offers. But how about the psychologic value of the dog? Is it nothing that he gives pleasure and companionship, stimulates affection and thoughfulness? Generally speaking, does not the boy or girl, brought up with such companionship, make a better man or woman? Doesn't the dog prevent insanity and suicide among those doomed to solitary occupations and especially to lives campaign of professional education brought him to trial? Or, without actual human companionship? If we adhere to a strict standard of utility, would we not send to the pound a good many human beings? For that matter, few of us are actually useful for more than a quarter of the time, and the majority seem mainly interested in being useful for the sake of enjoying the useless three-quarters of their time.

